# Oxygen Tension and Riboflavin Gradients Cooperatively Regulate the Migration of *Shewanella oneidensis* MR-1 Revealed by a Hydrogel-Based Microfluidic Device

**DOI:** 10.3389/fmicb.2016.01438

**Published:** 2016-09-20

**Authors:** Beum Jun Kim, Injun Chu, Sebastian Jusuf, Tiffany Kuo, Michaela A. TerAvest, Largus T. Angenent, Mingming Wu

**Affiliations:** ^1^Department of Biological and Environmental Engineering, Cornell UniversityIthaca, NY, USA; ^2^School of Chemical and Biomolecular Engineering, Cornell UniversityIthaca, NY, USA; ^3^Atkinson Center for a Sustainable Future, Cornell UniversityIthaca, NY, USA

**Keywords:** aerotaxis, motility, flavin, *Shewanella*, microfluidics, bioelectrochemical system (BES)

## Abstract

*Shewanella oneidensis* is a model bacterial strain for studies of bioelectrochemical systems (BESs). It has two extracellular electron transfer pathways: (1) shuttling electrons *via* an excreted mediator riboflavin; and (2) direct contact between the *c*-type cytochromes at the cell membrane and the electrode. Despite the extensive use of *S. oneidensis* in BESs such as microbial fuel cells and biosensors, many basic microbiology questions about *S. oneidensis* in the context of BES remain unanswered. Here, we present studies of motility and chemotaxis of *S. oneidensis* under well controlled concentration gradients of two electron acceptors, oxygen and oxidized form of riboflavin (flavin+), using a newly developed microfluidic platform. Experimental results demonstrate that either oxygen or flavin+ is a chemoattractant to *S. oneidensis.* The chemotactic tendency of *S. oneidensis* in a flavin+ concentration gradient is significantly enhanced in an anaerobic in contrast to an aerobic condition. Furthermore, either a low oxygen tension or a high flavin+ concentration considerably enhances the speed of *S. oneidensis.* This work presents a robust microfluidic platform for generating oxygen and/or flavin+ gradients in an aqueous environment, and demonstrates that two important electron acceptors, oxygen and oxidized riboflavin, cooperatively regulate *S. oneidensis* migration patterns. The microfluidic tools presented as well as the knowledge gained in this work can be used to guide the future design of BESs for efficient electron production.

## Introduction

**FIGURE 1:**
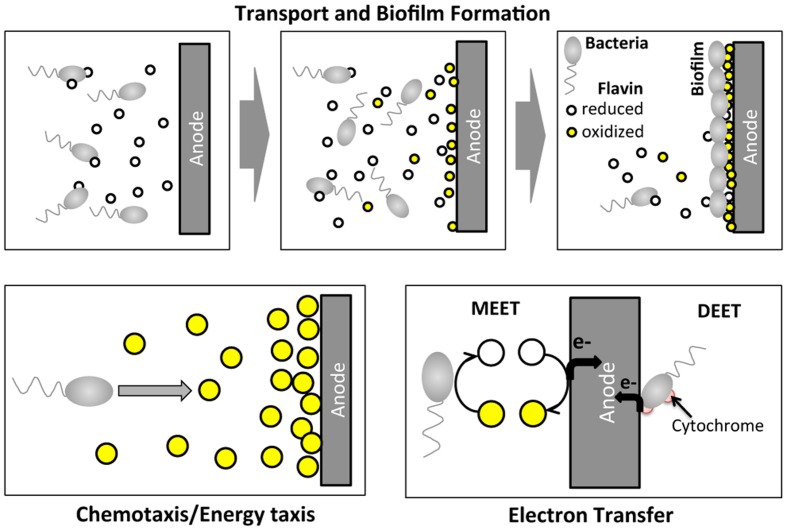
**Illustration of cell migration and electron transfer processes of *Shewanella oneidensis* MR-1 around an anode of a BES.** In BES operation, *S. oneidensis* bacteria migrate toward the anode and form a biofilm at the surface of the anode. At the same time, *S. oneidensis* bacteria secrete the reduced form of riboflavin while reducing their oxidized form. *S. oneidensis* potentially migrate toward high oxidized riboflavin concentration regions. At the anode, electron transfer occurs *via* two main mechanisms: mediated extracellular electron shuttles (MEET), riboflavins; and direct extracellular electron transfer (DEET) *via* cytochrome at the membrane.

Certain species of microbes (e.g., *Geobacter* spp.; *Shewanella* spp.; *Pseudomonas* spp.) have been found to transfer electrons from organic sources to extracellular electrodes, generating an electric current, which is the basis for a bioelectrochemical system (BES; Logan et al., 2006; Richter et al., 2008). BESs have received increasing attention recently because of their potential applications as microbial fuel cells (He et al., 2005; Rabaey and Verstraete, 2005; Logan et al., 2006) and biosensors (Li et al., 2011; Webster et al., 2014). A critical step in BES operation is the extracellular electron transfer process at the anode, which involves complex molecular and cellular transport (**Figure 1**). Within a BES, microbes migrate toward the anode and form a biofilm. At the same time, electron mediators (e.g., riboflavin or phenazine) secreted by the microbes assist the electron transfer process at the anode. *Shewanella oneidensis* MR-1 is a model microbial strain for BES, because it transfers electrons extracellularly *via* two paths: (1) a mediated extracellular electron transfer (MEET) using endogenously produced riboflavin; and (2) a direct extracellular electron transfer (DEET) *via* membrane bound cytochrome families (**Figure 1**) (Marsili et al., 2008; Li et al., 2012). Note that *S. oneidensis* is reported to produce reduced flavins *via* the Mtr pathway (Li et al., 2012).

The performance of a BES is influenced by many environmental factors, in particular, electron mediators including microbial secretions, oxygen, and electrodes (Rabaey et al., 2007; Fornero et al., 2008; Venkataraman et al., 2010). Recent studies using *S. oneidensis* as a model system have shown that oxygen tension critically regulates current production in a BES system; in particular, the current production is augmented in the presence of oxygen in a continuous-fed BES compared to anaerobic conditions (TerAvest et al., 2014). In the same study, electron mediator riboflavin is also reported to be implicated in the current production. A flavin-secretion-deficient mutant produced less current than the wild type *S. oneidensis*. This reduction is more pronounced in the micro-aerobic condition compared to that in the anaerobic condition, pointing to the direction that riboflavin and oxygen cooperatively regulate the current generation in BESs (TerAvest et al., 2014).

Despite the increasing interests in the development of BESs for applications as biosensors or microbial fuel cells, the electron production efficiency is far from optimized. This is in part due to the lack of basic understanding of the physical processes that take place within the BESs. Motility and directed migration of microbes have been shown to influence electron production in BESs (Harris et al., 2010, 2012). It has been reported that *S. oneidensis* MR-1 migrates toward various electron acceptors such as nitrate, fumarate, and Fe(III) under anaerobic condition using a classical swarm plate method or plug-in-pond assay (Nealson et al., 1995; Bencharit and Ward, 2005; Baraquet et al., 2009). Recent work by Harris et al. showed that *S. oneidensis* MR-1 swim faster either near MnO_2_ particles or an electrode with applied potentials, correlating the speed increase to the current production efficiency (Harris et al., 2010). Directional migration toward anaerobic electron acceptors has also been documented in *S. oneidensis*, requiring functional terminal reductase activities, mediated by an energy taxis mechanism (Baraquet et al., 2009). The current technology for cell motility and chemotaxis in the context of BES study is largely at macro-scale, and are not designed for providing complex microenvironment for cells.

Microfluidics is an enabling technology for exploring cellular behavior at single cell level in response to well-controlled environmental cues (Cheng et al., 2007; Ahmed et al., 2010). Microfluidics has a number of advantages in studying motilities of microbes in contrast to the conventional macro-scale devices. They include: (i) compatibility with optical microscopes, which allows for simultaneous monitoring of bacterial dynamics in space and real time; (ii) well defined chemical gradients enabling quantitative measurements (Diao et al., 2006; Kalinin et al., 2009, 2010); (iii) a fast and high throughput format. Here, miniaturization means a shorter time scale because diffusion is proportional to the length squared divided by the diffusion coefficient (The characteristic time for diffusion through a distance *L* is *L*^2^/2*kD*, where *k* is the spatial dimension and *D* is the diffusion coefficient); (iv) a large surface to volume ratio providing efficient extracellular electron transfer at electrode surfaces.

In this article, we study *S. oneidensis* migration in gradients of two electron acceptors, oxygen, and oxidized riboflavin using a newly developed microfluidic platform. We postulate that electron acceptors, oxidized riboflavin (flavin+) and oxygen, regulate the migration pattern of *S. oneidensis* synergistically. Because *S. oneidensis* transfer electrons both *via* secreted flavin as well as direct contact of the cell with the electrode, we argue that cell migration pattern within a BES critically controls the spatial distribution of bacteria within a BES, thus regulating electron transfer efficiency. Knowledge gained here can potentially be used for the design of BES optimized for efficient electron transfer at the anodes.

## Materials and Methods

### Cell Culture and Media

*Shewanella oneidensis* MR-1 (a gift from Dr. Tim Gardner, Boston University, Boston, MA, USA) was grown in Tryptone Broth (TB, 10.0 g/L of Bacto Tryptone, 5.0 g/L NaCl in 10 mM phosphate buffer at pH 7.1) in a shaker bath at 30°C, and with 150 rpm agitation. The overnight cultures were diluted in fresh TB medium (~25X) to OD_600_~0.05. Then, cells were harvested in the exponential growth phase when OD_600_ reached ~0.8. Cells were re-suspended twice (centrifuged at 1500 × *g* for 2 min) in M4 medium (Rosenbaum et al., 2010) containing 0.172% sodium lactate, 0.5 g/L Bacto Trypton, and 0.5 g/L Yeast Extract. A green fluorescent nucleic acid stain, SYTO^®^ 9 (Life Technologies, Carlsbad, CA, USA), was used at a concentration of 200 μM as instructed by manufacturer to confer fluorescence before aerotaxis and chemotaxis experiments. We compared the speed and tracks of cells with and without SYTO^®^ 9, and there was no difference during the experimental time period (up to 2 h). Note that SYTO^®^ 9 was used only for microfluidic migration studies, not for growth. *Escherichia coli* strain RP437 was a gift from Dr. Sandy Parkinson (University of Utah, Salt Lake City, UT, USA) (Parkinson, 1978) and the strain was transformed with pTrc-GFP carrying *gfpmut2* gene to produce green fluorescent protein (DeLisa et al., 2002). The cell culture protocol was similar to that for *S. oneidensis* except the plasmid induction step and the minimal medium composition. Arabinose (Sigma, St. Louis, MO, USA) was added to a final concentration of 0.2% when cell density reached OD_600_~0.2 to induce GFP expression. When OD_600_ reached ~0.8, cells were harvested and re-suspended twice (centrifuged at 1500 × *g* for 2 min) in M9 minimal medium (Sambrook et al., 1989).

### Microfluidic Device Fabrication and Assembly

**FIGURE 2:**
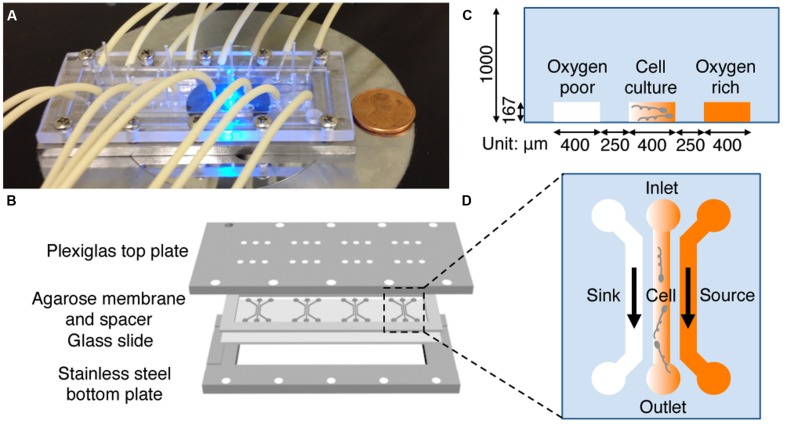
**Microfluidic setup and experimental design. (A)** A photograph of the microfluidic setup on the microscope stage. Tubing is used to perfuse media. **(B)** Illustration of the microfluidic setup. A 1 mm thick agarose gel membrane is patterned with four three-channel devices, placed on a 75 mm × 25 mm glass slide, surrounded by a 1 mm thick spacer, and sandwiched between a top Plexiglass plate and a bottom stainless steel plate with screws. The top plate has access holes for the tubing for medium transport. **(C,D)** Side **(C)** and top **(D)** view of one microfluidic device. Oxygen (or riboflavin) rich and buffer flow in source and sink channel, respectively. The gradient is generated in the center channel where bacteria are seeded. The channel dimension is 400 μm wide and 167 μm deep, the agarose ridge between channels is 250 μm wide.

A hydrogel based microfluidic device (**Figure 2**) was designed and constructed to provide a stable, linear chemical gradient in a microfluidic channel where cells were introduced. Details of the device can be found elsewhere (Cheng et al., 2007). Briefly, four three-channel device patterns were fabricated on a silicon master using the standard photolithography technique at the Cornell NanoScale Science and Technology Facility (CNF). The hydrogel membrane with the device pattern was molded off the silicon master. More specifically, we first poured 3% hot agarose gel (0.3 g agarose in 10 mL PBS) onto the silicon master surrounded by a polydimethylsiloxane (PDMS) spacer of 1 mm thickness, and then gently peeled it off once the membrane was gelled at room temperature. The hydrogel membrane was then soaked in an appropriate medium for at least 30 min (can be stored for up to a week in a refrigerator) before being used. In a typical experiment, the patterned membrane was sandwiched between a plastic manifold and a glass slide (75 mm × 25 mm), supported by a stainless steel frame (**Figure 2B**). The plastic manifold was pre-built with all the inlets and outlets for the microfluidic channels. The flows in the side channels were provided by a peristaltic pump (Watson Marlow 205S with eight parallel lines; Watson Marlow, Wilmington, MA, USA). Four identical three-channel devices are patterned in one membrane, which allow for four parallel experiments to be conducted at the same time using cells from the same batch.

### Oxygen Tension Control and Measurement

To prepare oxygen depleted medium, we used a 50 mL glass bioreactor unit (Cellstation, Rockville, MD, USA) equipped with a gas-sparging unit and head-space outlet with a filter. This protocol had been used successfully for depleting oxygen in medium for obtaining volumetric oxygen transfer coefficients in a bioreactor previously, and the near zero oxygen concentration of nitrogen saturated medium had been verified using an oxygen probe (Kim et al., 2012). For each operation, 20 mL of fresh medium was placed into the bioreactor, and then the system was sparged with pure nitrogen gas for at least 20 min. The nitrogen-saturated (oxygen poor) medium was pumped out through the harvest port of the bioreactor using a tubing and a peristaltic pump. All tubing was made of biocompatible, low gas-permeable materials (Cole-Parmer, Vernon Hills, IL, USA). Here, the oxygen concentration of the nitrogen saturated medium is considered to be [O_2_] = 0. To prepare an oxygen rich medium, we used the medium left in the normal atmosphere without sparging. The air-saturated medium had oxygen concentration of [O_2_] = 8.56 mg/L at 23°C (a standard concentration of oxygen from EPA).

To measure the oxygen concentration, we used an oxygen-sensitive fluorescent dye, ruthenium tris(2,20-dipyridyl) dichloride hexahydrate (RTDP, Sigma, St. Louis, MO, USA). We note that the verification of oxygen gradients using RTDP was done with media only, without cells. The fluorescence of RTDP is known to be quenched in the presence of oxygen (Adler et al., 2010), and the relationship of the monitored fluorescence intensity, *I*, and the oxygen concentration, [O_2_], is described by a Stern-Volmer equation: I_0_/I = 1 + K_q_[O_2_]. Here, *I*_0_ is the fluorescence intensity at [O_2_] = 0 mg/L, and *K*_q_ is the quenching constant. The quenching constant was obtained experimentally using the maximum and minimum fluorescence intensity measured in air saturated medium with [O_2_] = 8.56 mg/L at 23°C, and the nitrogen saturated medium with [O_2_] = 0 mg/L, respectively. The quenching constant in our medium using an entire image set (41 images) was averaged to be ~3.66, in contrast to the reported values of 2.25 for solutions of RTDP in water (Polinkovsky et al., 2009). This difference can be attributed to the fact that the pixel values recorded by the camera have a non-zero background component with respect to the based fluorescence intensity. Because of this, the measured oxygen concentration is a relative value, not an absolute one.

### Riboflavin+ and/or Oxygen Concentration Gradient Generation and Characterization

Chemical concentration gradients are generated using a hydrogel based three-channel microfluidic device (**Figure 2**). Briefly, the two side channels (sink and source) are used to flow media and chemo-attractant, respectively, and the gradient is established in the middle (or cell) channel *via* diffusion.

To establish a riboflavin+ (Sigma-Aldrich, stored at 40 μM after filter sterilization with PTFE) gradient, medium with riboflavin+ flows in the source channel while plain medium flows through the sink channel. A riboflavin+ gradient is generated in the center channel. The microfluidic chemical gradient generator has been used successfully to generate chemical concentration gradients as detailed elsewhere (Diao et al., 2006; Cheng et al., 2007). The validation and calibration of the riboflavin+ concentration gradient is discussed in Supplementary Figure S1. We note that care must be taken to achieve anaerobic conditions for riboflavin+ gradient experiments. Therefore, both media with and without 50 nM riboflavin+ were placed in the bioreactor units and the solution was purged with pure nitrogen for at least 20 min. The entire microfluidic setup was additionally covered with a plastic container connected to pure nitrogen to ensure anaerobic conditions.

**FIGURE 3:**
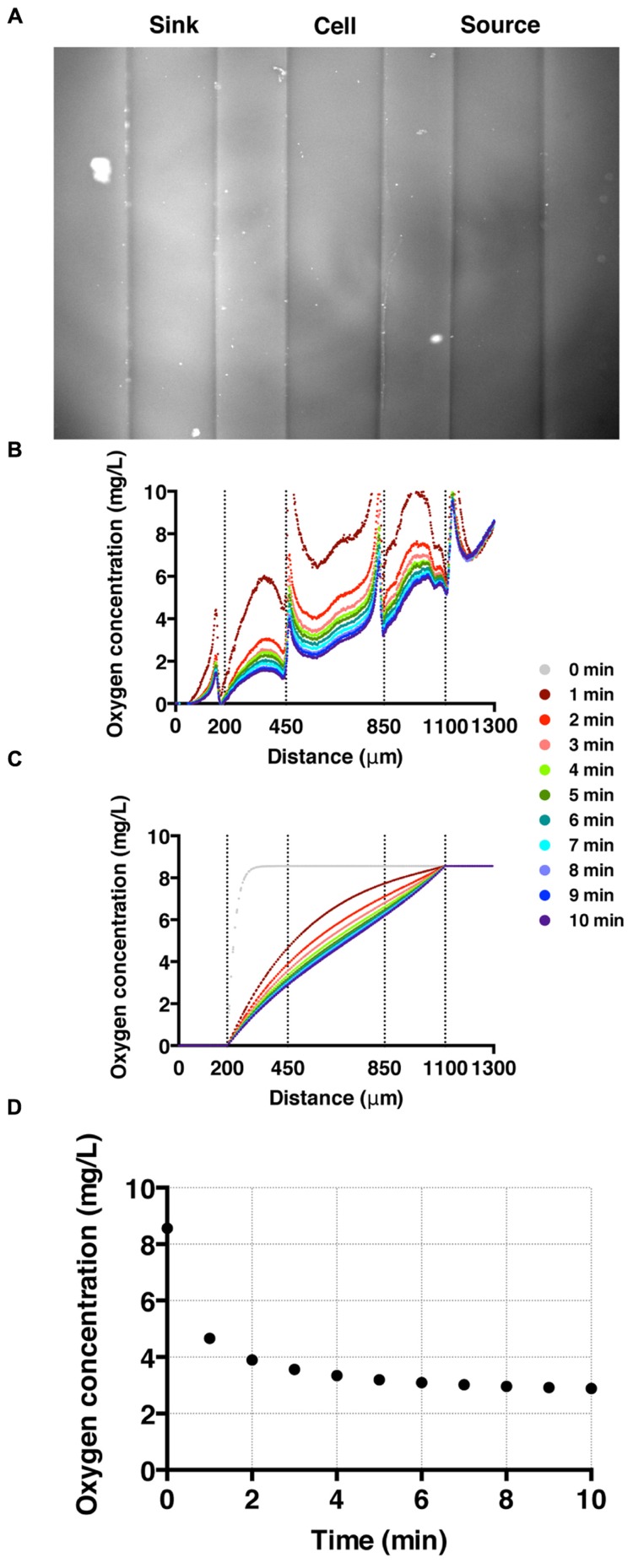
**Oxygen gradient generation and calibration. (A)** A fluorescent image of the three channels with oxygen-rich and oxygen-poor media in the source and sink channel, respectively. The fluorescence intensity corresponds to the fluorescence quenching due to RTDP. **(B)** A time sequence of oxygen concentration profiles across all three channels over time. Here, *t* = 0 corresponds to the time when oxygen-rich and oxygen-poor media are introduced into the side channels. The vertical dotted lines mark the locations of channel walls, and the peaks near the dotted lines are artifacts of the agarose gel walls. **(C)** A time sequence of oxygen concentration profiles. Results of simulation obtained using COMSOL multiphysics computation software. **(D)** The time evolution of the oxygen concentration at the center of the cell channel as a function of time in experiment. It demonstrates that it takes about 3 min for the oxygen gradient to reach to a steady state.

To establish and calibrate an oxygen concentration gradient, we flowed nitrogen-saturated and air-saturated media through sink and source channels, respectively, and imaged all three channels for 20 min with an interval of 15 s. We confirmed that the oxygen gradient was reversed by switching the tubing for the sink and source channels. High fluorescence intensity in the sink channel (left) is observed as oxygen poor medium is pumped through the channel; while low florescence intensity is shown in the source channel where the oxygen rich medium flows (**Figure 3A**). The time-evolution of the oxygen concentration profile across the three channels was also monitored (**Figure 3B**). These results agree well with the computed oxygen concentration profiles over time using COMSOL Multiphysics software (Burlington, MA, USA) (Haessler et al., 2009, 2011; Chang et al., 2013). In the numerical calculation, we used a diffusion coefficient of 2 × 10^-5^ cm^2^/s (Adler et al., 2010). The time needed to reach a steady-state oxygen gradient from the simulation (**Figure 3C**) is similar to that of the experiment (**Figure 3B**), which is about 3 min (**Figure 3D**).

### Imaging and Data Analysis

An Olympus epi-fluorescence microscope (IX51, Center Valley, PA, USA) with a 20× objective lens (UPlan; numerical aperture, 0.5), EXFO X-Cite 120 Fluorescence Illumination System (EXFO, Ontario, Canada), and a CCD camera (Cascade 512B camera; Photometrics, Tucson, AZ, USA) was used for all the experiments reported here. For visualizing cells with green fluorescent stain, we used a FITC/EGFP (λ _EX_ = 455–500 nm/λ _EM_ = 510–560 nm; Chroma Technology Inc., Bellows Falls, VT, USA) filter cube. For imaging oxygen concentration *via* RTDP, we used a Texas-red filter (λ _EX_ = 542–582 nm/λ _EM_ = 604–644 nm).

**FIGURE 4:**
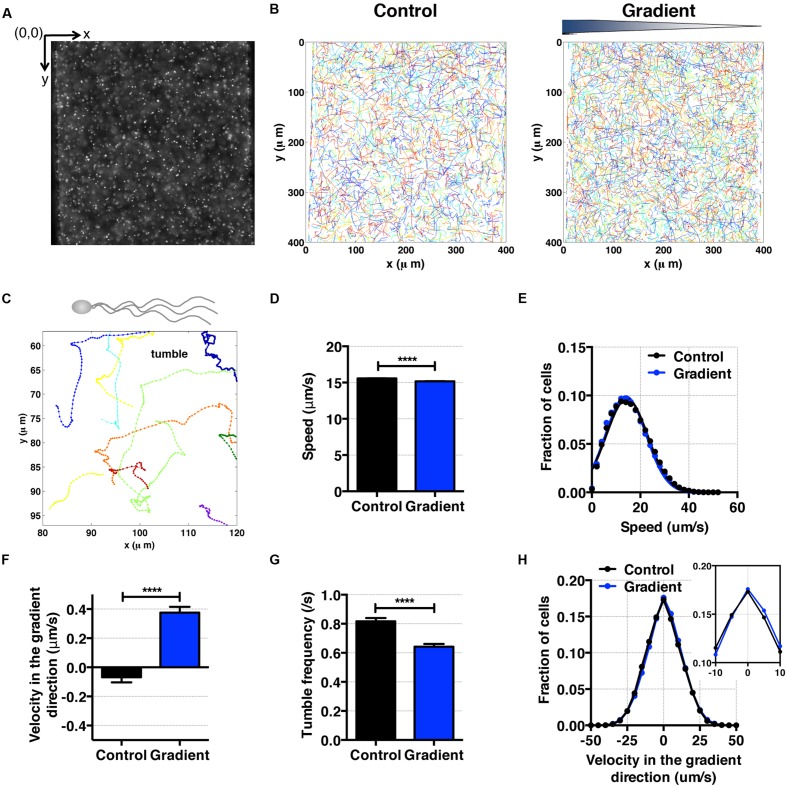
***E. coli* bacteria motility and aerotaxis in an oxygen gradient. (A)** A fluorescence image of *E. coli* in a microfluidic channel (channel width: 400 μm). **(B)** Cell tracks of *E. coli* across the channel in the absence (control) and the presence of an oxygen gradient. **(C)** A single *E. coli* cell has multiple flagella, showing the characteristic run-and-tumble motility. Each colored line is a single bacteria track. **(D)** Average *E. coli* swimming speed in the absence and presence of oxygen gradients (9.51 mg/L/mm) indicates that the average speed is not altered much by the presence or absence of the oxygen (15.58 ± 0.02 μm/s vs. 15.17 ± 0.03 μm/s, mean ± S.E.). **(E)** Distribution of instantaneous speed in the absence (control) and the presence of an oxygen gradient. The distribution shift is negligible. **(F)** Average *E. coli* swimming velocity along the oxygen gradient (+*x*-axis). Directional migration along the oxygen gradient is shown by the positive average bacterial velocity along the oxygen gradient direction. The number of instantaneous speed or velocity for computing average values is 112,607 for the control and 87,973 for the gradient case. **(G)** Tumble frequency (s^-1^) decreases in the presence of oxygen gradient. **(H)** Distribution of instantaneous x-velocity in the absence (control) and the presence of an oxygen gradient. There is a distribution shift toward the positive x-velocity, indicating that there is an aerotactic behavior in *E. coli*. Student *t*-test was applied and the results are represented with a significant difference of *p*-value less than < 0.0001 for **(D),** **(F),** and **(G)** (^∗∗∗∗^: *p* < 0.0001).

**FIGURE 5:**
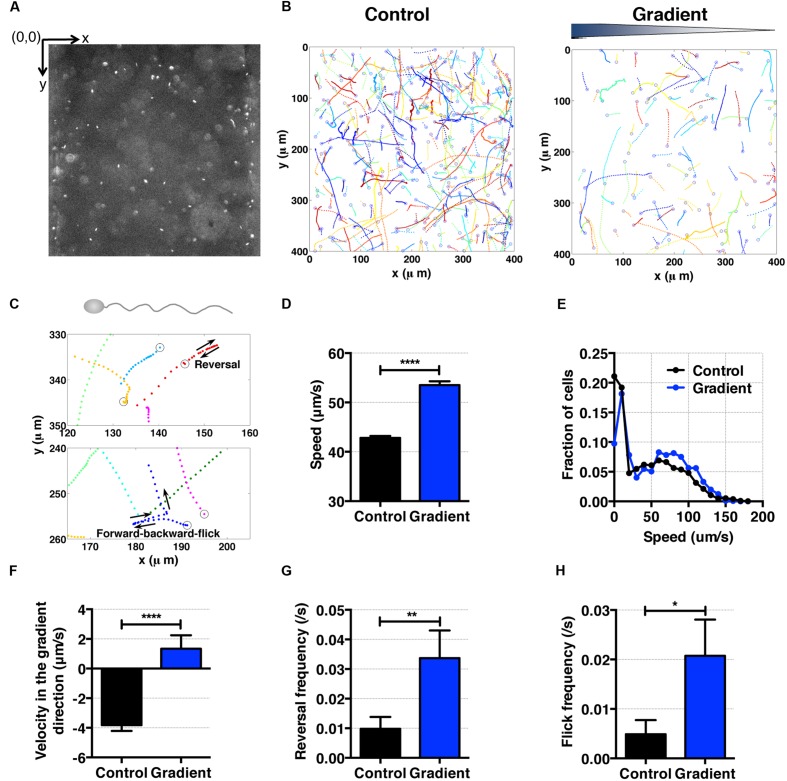
***S. oneidensis* motility and aerotaxis in an oxygen gradient. (A)** A fluorescence image of *S. oneidensis* in a microfluidic channel (channel width: 400 μm). **(B)** *S. oneidensis* cell tracks across the channel in the absence (control) and presence of an oxygen gradient. Open circles represent the beginning of a track. **(C)** Characteristic motility pattern of single flagellated bacterium. A reversal occurs when the bacterium switches its swimming direction by 180°C. A forward-backward-flick occurs when the bacterium reverses its direction and then changes its direction shortly along a direction that is almost perpendicular to its original swimming direction. The open circle marks the beginning of the track. **(D)** Average *S. oneidensis* swimming speed in the absence and presence of oxygen gradients. The presence of the oxygen gradient considerably enhances *S. oneidensis* swimming speed. **(E)** Distribution of instantaneous speed in the absence (control) and the presence of an oxygen gradient. The distribution shifts toward higher speed and widens in the presence of the gradient. **(F)** Average *S. oneidensis* swimming velocity along the oxygen concentration gradient direction (*x*-axis) in the absence and presence of oxygen gradients. *S*. *oneidensis* displays a directional migration along oxygen gradient. The number of instantaneous speed or velocity for computing average values is 10,094 for control and 2,949 for gradient case. We note that there is a drift velocity of ~2 μm/s in the control experiment. Aerotaxis was confirmed in three independent experiments. **(G)** Reversal frequency increases in the presence of oxygen gradient. **(H)** Flick frequency increases in the presence of oxygen gradient. Student *t*-test was applied and the results are represented with a significant difference of *p*-value less than < 0.0001 for **(D)** and **(F)** (^∗∗∗∗^: *p* < 0.0001), between 0.001 and 0.01 for **(G)** (^∗∗^: 0.001 < *p* < 0.01), and between 0.01 and 0.05 for **(H)** (^∗^: 0.01 < *p* < 0.05).

For cell motility and aerotaxis experiments, we imaged the middle portion of the center channel at 10 min after the media were introduced into the side channels. A movie of 500 frames was taken at a frame rate of 30 fps (IPLab imaging software, BD, Franklin Lakes, NJ, USA). These movies were post-processed to obtain cell positions and subsequent motility parameters using an in-house MATLAB program (Liao et al., 2007). Using images taken (**Figures 4A** and **5A**), individual cell tracks were generated (**Figures 4B** and **5B**). Because the bacteria tend to swim near the wall (Berke et al., 2008), we analyzed bacteria swimming in the central portion of the channel to avoid the wall effect. More specifically, the original image covers the total channel width, which is 400 μm, however, we use only the middle portion of the image, 240 μm (width) × 410 μm (height) for the case *E. coli*, and 320 μm (width) × 410 μm (height) for the case of *S. oneidensis*, for further data analysis. The tracks of non-motile cells were excluded from further analysis. The standard deviation of positions of a non-motile cell track is equal or less than 3 pixels (or 2.4 μm) for *E. coli* and 8 pixels (or 6.4 μm) for *S. oneidensis*.

**FIGURE 6:**
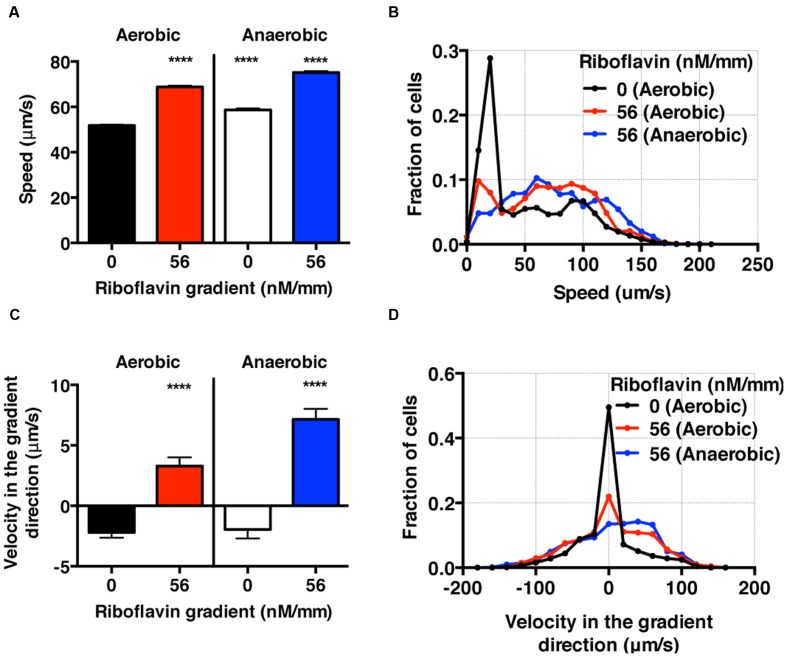
***S. oneidensis* motility and chemotaxis in a riboflavin gradient. (A)** Average *S. oneidensis* swimming speed in control and 56 nM/mm riboflavin gradient under aerobic and anaerobic conditions. The presence of riboflavin considerably enhances *S. oneidensis* swimming speed in both aerobic and anaerobic conditions. **(B)** Distribution of instantaneous speed shifts toward higher speed and widens in the presence of riboflavin gradients. **(C)** Average *S. oneidensis* swimming velocity along the riboflavin concentration gradient (56 nM/mm) direction under aerobic and anaerobic conditions. *S*. *oneidensis* displays a directional migration along the riboflavin gradient, in both aerobic and anaerobic conditions. The chemotactic tendency is enhanced in anaerobic condition compared to in aerobic condition. **(D)** Distribution of instantaneous velocity shifts toward higher positive values and widens in the presence of riboflavin gradients. The number of instantaneous speed or velocity for computing average values is 8,241 for aerobic control, 5,126 for aerobic gradient, 4,479 for anaerobic control, and 3,938 for anaerobic gradient case. Student *t*-test was applied and the results are represented with a significant difference of *p*-value less than < 0.0001 (^∗∗∗∗^: *p* < 0.0001).

For characterizing cell motility and aerotaxis, we computed the instantaneous speed and the average instantaneous velocity in the x-direction (gradient direction) using the cell tracks that were obtained as described above. Here, instantaneous speed is defined as the length of the cell track traveled by a cell between two consecutive images divided by time (33 ms = 1/30 s). The instantaneous velocity is defined as the difference of a cell position along the *x*-axis of two consecutive images divided by time. Typically, a 500-frame movie provides more than 2000 (for *E. coli*) or 300 (for *S. oneidensis*) tracks for data analysis. In addition to the chemotactic parameters listed above, the chemotactic migration coefficient (CMC) was also calculated from the cell tracks (Kalinin et al., 2009). CMC is the average x position of all the cells tracked with respect to the center of the channel. The channel width used for *E. coli* is 240 μm, so the *CMC = [mean(x_i_)-x_c_]/120 μm* where *x*_i_ is the individual cell position along *x*-axis, and *x*_c_ is the *x* coordinate of the center of the channel. We note that the channel width used for *S. oneidensis* is 320 μm. Here, CMC = 1 means that all the cells have migrated and reached the side of channel with high chemo-attractant concentration. CMC = 0 means that cells are executing random motion, and that no chemotactic motion is observed. For a typical set of experiments, we ran four parallel experiments on the same chip: two experiments with no gradients, and two with gradients. Three sets of independent experiments were carried out and analyzed. We present one typical experimental result for each cell strain (**Figures 4–6**). We also analyzed individual cell tracks to obtain tumble or reversal/flick frequency. For the analysis of tumble or reversal/flick frequency, we utilized all the tracks from three experiments to obtain as many tumbles or reversal/flicks as possible. The detailed information for tumble or reversal/flick frequency analysis from three experiments is provided in Supplementary Tables S1 and S2.

## Results and Discussion

### *Escherichia coli* Aerotaxis Using Hydrogel Based Microfluidic Device

As an experimental control, we first studied *E. coli* aerotaxis and aerokinesis in oxygen gradients for testing the newly developed microfluidic oxygen concentration gradient generator. *E. coli* cells swim by rotating 6–7 flagella, and are known to be aerotactic in oxygen gradients (Rebbapragada et al., 1997; Greer-Phillips et al., 2003; Adler et al., 2012). Here, we confirmed previous findings. Using the tracked single cell trajectories, we observed the characteristic run and tumble motion of single *E. coli* cells (**Figures 4A–C**). The average cell speed remained the same when the average oxygen conc. was reduced from 8.56 to 4.28 mg/mL (**Figure 4D**), 15.58 ± 0.02 (control) and 15.17 ± 0.03 μm/s (oxygen gradient). The distributions of instantaneous speed in both cases were indistinguishable (**Figure 4E**). This indicates that the aerokinesis (speed change under an oxygen gradient) is negligible in *E. coli*. In contrast, cell velocity along the gradient was significantly increased in the presence of oxygen gradients (**Figures 4F,H**). The aerotactic tendency, measured by CMC also supports that *E. coli* shows aerotaxis behavior, with 0.028 ± 0.002 vs. 0.005 ± 0.002 (gradient vs. control). These experimental results are consistent with previous work in *E. coli* aerotaxis (Bibikov et al., 1997; Rebbapragada et al., 1997; Adler et al., 2012), validating the hydrogel based microfluidic device for studies of aerotaxis.

To further characterizing *E. coli* aerotaxis, we analyzed tumble frequencies to determine if the oxygen gradients affect the tumbling rates, as they do in *E. coli* chemotaxis. We found that the tumble frequency decreased from 0.82 ± 0.02 s^-1^ in the control to 0.64 ± 0.02 s^-1^ in an oxygen gradient, which confirms that the run length change is a dominant mechanism in *E. coli* aerotaxis. This interpretation is consistent with the results from *E. coli* chemotaxis (Berg and Brown, 1972), where *E. coli* bacteria lengthen their run length when swimming up a chemoattractant gradient. We note that the time between two consecutive tumbling (1/0.82 s^-1^ = 1.2 s), is consistent with the reported value in literature (0.86 ± 1.18 s) for *E. coli* chemotaxis (Berg and Brown, 1972).

Using the hydrogel based microfluidic device, we generated results that are in agreement with the previously reported aerotactic behavior of *Escherichia coli* (Adler et al., 2012). The unique feature of the device is that it enables a 3D cell migration study in the presence of dissolved gas gradients. Previous hydrogel based microfluidic device uses alginate as a base material, as a result, cells are immobilized within a cross linked alginate when subjected to dissolved gas gradients (Choi et al., 2012). In contrast to the PDMS (polydimethylsiloxane, a gas permeable material) based O_2_ gradient generator (Mehta et al., 2007; Skolimowski et al., 2010; Brennan et al., 2014), our hydrogel-based microfluidic device is less prone to air bubble and evaporation problems because the entire device is surrounded by mostly medium (Adler et al., 2012). Note that the base material of our device, the agarose gel, contains 97% of medium. In the PDMS device case, the cell embedded medium has to be in continuous flow or the device needs to have a hydration layer to avoid evaporation and air bubbles. Furthermore, the hydrogel-based device can be easily extended to include different oxygen concentration gradients when gasses of various oxygen concentrations are used during sparging phase, as well as gradients of other biologically relevant gas, such as hydrogen or carbon dioxide.

### *Shewanella oneidensis* Bacteria Display Motility of Single Flagellated Bacteria, and Are Aerotactic and Aerokinetic in Oxygen Gradients

*Shewanella oneidensis* is a single flagellated and facultative anaerobe (Venkateswaran et al., 1999; Paulick et al., 2009). With a single flagellum, it swims by rotating the flagellum along its long axis (Paulick et al., 2009). Using tracked bacterial trajectories, we observed distinct migration patterns of single-flagellated bacteria, including straight run, reversal (**Figure 5C**, top panel), and forward-backward-flick (**Figure 5C**, bottom panel). This is consistent with the previous observation for the swimming behavior of single-flagellated bacteria such as *Vibrio alginolyticus* (Stocker, 2011; Xie et al., 2011). *V. alginolyticus* was reported to reverse its run direction when the rotation of the single flagellum switched from clockwise to counter-clockwise or *vice versa* (Goto et al., 2005). Furthermore, the flick motion was initiated at the base of flagellum where the flagellum functioned as a rudder (not only as a propeller; Stocker, 2011; Xie et al., 2011). We observed with *S. oneidensis* an angle of flick perpendicular to the forward-backward axis in most cases, which was consistent with those of *Shewanella* sp. (Bubendorfer et al., 2014) and *V. alginolyticus* (Xie et al., 2011).

To characterize the aerotactic behavior of *S. oneidensis*, we calculated the average cell speed and cell velocity along the oxygen gradient. We found that *S. oneidensis* swims faster in oxygen gradient in contrast to the control (no gradient, air-saturated), with an average speed of 53.51 ± 0.79 μm/s versus 42.81 ± 0.44 (**Figure 5D**) or a percentage change of 20%. The average speed distribution further affirms the increase in motility in the low oxygen tension case (**Figure 5E**). The results for average velocity along the gradients show that *S. oneidensis* cells migrate toward the higher concentration of oxygen, demonstrating aerotactic behavior (**Figure 5F**). The computed chemotactic index, the CMC value (-0.018 ± 0.010) under the oxygen gradient is not distinctively different from that (-0.024 ± 0.006) under uniform oxygen environment. We note that our device has a systematic bias toward the -*x* direction (+*x* direction is the chemical gradient direction) in all the measurements. This bias is more pronounced in the case of fast moving cells (*S. oneidensis, or* **Figure 5F**) in contrast to slow moving cells (*E. coli* or **Figure 4F**). To further understand the mechanism of directional changes under oxygen gradient, we calculated frequencies of reversal and forward-backward-flick events using the tracks in **Figure 5B**. The change in frequency of reversal was found to contribute the congregation of *S. oneidensis* near insoluble electron acceptors (Harris et al., 2012). We found that the reversal frequencies increased from 0.080 ± 0.012 to 0.16 ± 0.01 s^-1^ (**Figure 5G**), and the forward-backward-flick frequencies increased from 0.028 ± 0.008 to 0.064 ± 0.008 s^-1^ under oxygen gradients (**Figure 5H**). It remains to be investigated whether both speed increase and directional change contribute to their directional migration along oxygen gradients, which is different from the case of *E. coli* aerotaxis. We note that the forward-backward-flick frequency might be underestimated because the average tracking time per cell is ~0.8 s, which is much shorter than the average time between flicks (on the order of 10 s). We also note that the reversal frequency was ~0.02 s^-1^, which is lower than the reversal frequency (0.02~1.2 s^-1^) reported by Harris et al. (2012).

In contrast to the aerotaxis of *E. coli*, the aerotactic behavior of *S. oneidensis* are largely unexplored. We know that both Aer and Tsr sensor/transducer proteins are involved with *E. coli* aerotaxis, not through the direct receptor–ligand binding, but through the changes in the redox status of the electron transport system and the transmembrane proton gradient in a metabolism-dependent manner (Greer-Phillips et al., 2003; Baraquet et al., 2009; Harris et al., 2012). The latter behavior is also termed as “energy taxis” in literature and its stimuli include terminal electron acceptors such as oxygen, nitrate, and redox-active chemicals. Although a homolog of *aer* gene was identified with *S. oneidensis* (Beliaev et al., 2005), the detailed molecular mechanism of aerotaxis of *S. oneidensis* remains to be explored (Stocker et al., 2008; Xie et al., 2011).

### *S. oneidensis* Cell Chemotactic Behavior toward High Concentrations of Riboflavin Is Enhanced Significantly in Anaerobic in Contrast to Aerobic Conditions

In the context of BESs, there are competing metabolic processes that govern the use of different electron acceptors depending on the oxygen levels: in an aerobic condition, oxygen serves as the electron acceptor, while in an anaerobic condition, the oxidized form of riboflavins may serve as electron acceptors (Marsili et al., 2008). In this context, we studied the migration pattern of *S. oneidensis* under riboflavin+ gradients in the presence and absence of oxygen gradients.

Using the bacterial tracks, we found that the speed of *S. oneidensis* is enhanced under the riboflavin+ gradient (56 nM/mm or average riboflavin concentration of 25 nM) in contrast to the control in both aerobic (8.56 mg/L) and anaerobic condition (**Figures 6A,B**). It is also interesting to note that the speed of *S. oneidensis* is enhanced under the anaerobic condition, compared to the aerobic condition, with or without riboflavin+. The directional migration toward high riboflavin+ concentrations is distinctly seen in the average velocity along the flavin+ gradient direction in contrast to the control in the case of aerobic condition (**Figure 6C,D**). Furthermore, this chemotactic tendency is enhanced significantly in the anaerobic condition compared with the aerobic condition (**Figure 6C,D**).

Here, we presented the synergistic roles of flavin+ and oxygen tension in the chemotaxis of *S. oneidensis* for the first time. We found that the chemotactic behavior of *S. oneidensis* is significantly enhanced in the anaerobic over the aerobic condition. We conjecture that the lack of electron acceptors in anaerobic condition may cause the enhanced chemotactic activities of *S. oneidensis* in riboflavin+ gradients. We also note that the endogenous riboflavin production is known to be higher in the aerobic condition (Von Canstein et al., 2008; TerAvest et al., 2014), which may mask the bacteria’s sensitivity to exogenously imposed riboflavin+ gradient. This information is important in BES because the roles of oxygen in the optimization of microbial fuel cells are still under debate (Biffinger et al., 2009).

The presented results that electron acceptors are chemoattractant to *S. oneidensis* are consistent with previous reports. *S. oneidensis* motility and chemotaxis in the presence of electron acceptors have been studied using metals or metal oxides such as MnO_2_ particles in detail (Harris et al., 2010, 2012). Cell speed was found to increase in the vicinity of the MnO_2_ particle. In a second work, *S. oneidensis* chemotaxis and motility have been studied using a population level swarm plate assay (Li et al., 2012) in which riboflavin was found to be chemoattractant. The presented work here is the first extensive study on how cells respond to two electron acceptors at a single cell level.

## Concluding Remarks

In this paper, we present a study of *S. oneidensis* in oxygen and/or riboflavin gradients using a hydrogel gel based microfluidic device. Our work demonstrates that *S. oneidensis* exhibits the directional migration toward either high oxygen or high riboflavin+ concentration area, indicating that both electron acceptors are attractant to *S. oneidensis.* Furthermore, the chemotactic tendency in riboflavin+ alone is augmented significantly in anaerobic condition in contrast to aerobic condition. These results highlight the importance of dual electron acceptors in regulating motility and chemotaxis of *S. oneidensis*.

Results of the presented work can impact future BES research in two ways. One is on the technology front introducing microfluidic technology for BES research. The current main research tool for BES is a bioreactor in a jar, which is not suitable for large scale environmental parameter optimization. A microfluidic BES, integrating electrodes into the hydrogel based microfluidic platform, will allow for a systematic and fast environmental parameter screening, including endogenous/exogenous electron mediators, nutrient condition, co-culture, and oxygen tension, for optimal electron transfer. This is important because we know that the performance of the existing BES is far from the theoretical electron transfer limit, and environmental factors critically regulate electron transfer efficiency (Schroder, 2007). The second is on the science front. A mechanistic understanding of how endogenous/exogenous electron mediators and oxygen direct cell migration will allow us to predict cell spatial distribution in a given BES architecture (e.g., shape and size of the electrodes), leading to a new class of BES design for optimal electron transfer. Future work will require close collaborations among microbiologists, chemical engineers, and environmental engineers.

## Author Contributions

BK, MT, LA, and MW designed the work. BK, IC, SJ, TK, and MT conducted the experiments. BK, IC, SJ, and TK analyzed the data. BK, IC, MT, LA, and MW wrote the manuscript.

## Conflict of Interest Statement

The authors declare that the research was conducted in the absence of any commercial or financial relationships that could be construed as a potential conflict of interest.
