# Supplementary material for: Oxygen Tension and Riboflavin Gradients Cooperatively Regulate the Migration of Shewanella oneidensis MR-1 Revealed by a Hydrogel-Based Microfluidic Device
Source: Front Microbiol. 2016 Sep 20;7:1438. doi: 10.3389/fmicb.2016.01438 (PMC5028412; doi:10.3389/fmicb.2016.01438)

Supplementary Material.

Tables S1 and S2 provide detailed information on data analysis results of individual tracks generated using Matlab from three independent experiments. Track duration represents the time of an individual track. We counted the number of occurrences for each behavior (tumble, reversal, or reverse-flick), and the frequency was defined as the ratio of the total number of occurrences to the total track duration. The standard error was calculated based on error ~ $\sqrt{\text{number}}$. For example, the frequency of tumble in control of trial #1 is 328/(400×1.30 s) = 0.631 s^{-1} and the standard error is $\sqrt{328}$/(400×1.30 s) = 0.034 s^{-1}.

Table S1. ***E. coli*** **cell tumble analysis from aerotaxis experiments.**

Trial	Condition	Cell #	Mean track duration (s)	# of tumbles	Tumble frequency (s^{-1})
#1	Control	400	1.30	328	0.631±0.034
	Gradient	400	1.16	246	0.531±0.034
#2	Control	400	1.37	509	0.929±0.041
	Gradient	400	1.58	386	0.611±0.031
#3	Control	400	1.31	465	0.885±0.040
	Gradient	400	1.35	418	0.773±0.037
Total	Control	1200	1.33	1302	0.817±0.022
	Gradient	1200	1.36	1050	0.642±0.020

Table S2. ***S. oneidensis*** **MR-1 cell reversal and flick analysis from aerotaxis experiments.**

Trial	Condition	Cell #	Mean track duration (s)	# of reversals	Reversal frequency (s^{-1})	# of flicks	Flick frequency (s^{-1})
#1	Control	287	0.778	1	0.004±0.004	0	0
	Gradient	42	0.667	2	0.071±0.050	1	0.036±0.036
#2	Control	239	0.999	3	0.013±0.007	2	0.008±0.006
	Gradient	292	0.690	9	0.045±0.015	5	0.025±0.011
#3	Control	153	0.982	2	0.013±0.009	1	0.007±0.007
	Gradient	229	0.683	2	0.013±0.009	2	0.013±0.009
Total	Control	679	0.902	6	0.010±0.004	3	0.005±0.003
	Gradient	563	0.685	13	0.034±0.009	8	0.021±0.007

Figure S1. Riboflavin gradient generation and calibration. (A) A time sequence of riboflavin concentration profiles across all three channels over time. Here t = 0 corresponds to the time when 40 μM riboflavin and PBS buffer are introduced into the side channels. **(B)** A time sequence of riboflavin concentration profiles obtained using COMSOL multiphysics computation software.

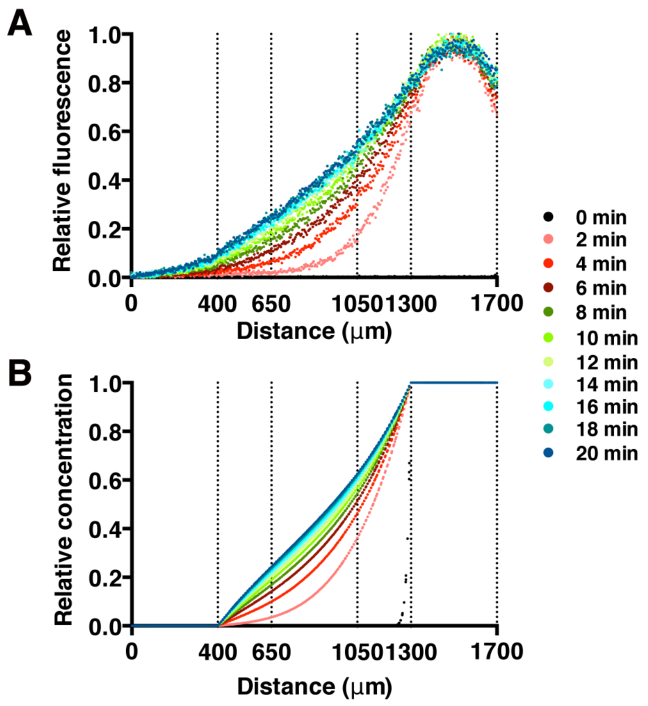

Supplement: Supplementary file 1 [file Data_Sheet_1.PDF]
